# Meralgia Paresthetica: A Report of a Rare Case

**DOI:** 10.7759/cureus.60440

**Published:** 2024-05-16

**Authors:** Catarina Vasconcelos Fonseca, João Dias, Tiago Pimenta, André Varandas Borges, Maria João Cotter

**Affiliations:** 1 Physical Medicine and Rehabilitation, Centro Hospitalar de Trás-os-Montes e Alto Douro, Vila Real, PRT

**Keywords:** physiatry, ultrasound-guided, intervention, polytrauma, meralgia paresthetica

## Abstract

Meralgia paresthetica (MP) is a painful condition caused by damage or constriction of the lateral femoral cutaneous nerve (LFCN). This entrapment condition typically arises due to various factors, including trauma, pelvic tumors, external compression from belts or snug attire, and weight gain. The prognosis is generally favorable since most cases are self-limiting or respond to conservative treatment. We present the case of a 53-year-old overweight man, with no relevant medical history, who was a victim of a traffic accident in October 2023 which resulted in polytrauma, according to the Case Reports (CARE) checklist. The main complaint of the patient was tingling of the left thigh, with dysesthesia to gentle rubbing along the anterolateral surface. After a diagnostic study, a diagnosis of post-trauma MP was thus established, probably due to seat-belt compression of the LFCN, and physiatric treatment was initiated. With the assistance of ultrasound, a large hematoma was seen, above the inguinal ligament with drainage of 140ccc of serosanguineous fluid with resolution of the symptoms. This case emphasizes the importance of a physiatry consultation for a correct diagnosis and focuses on the main complaint of a polytrauma patient.

## Introduction

The lateral femoral cutaneous nerve (LFCN) of the thigh originates from the lumbar L2 and L3 nerve roots, joining the lumbar plexus, and then piercing through the psoas muscle and, usually, exiting the pelvis posterior to the inguinal ligament and medial to the anterior superior iliac spine (ASIS). Below the inguinal ligament, the LFCN commonly splits into both anterior and posterior branches. This nerve is a purely sensory nerve supplying sensation to the anterolateral surface of the thigh [1].

Meralgia paresthetica (MP) is a painful condition caused by damage or constriction of the LFCN as it passes through the inguinal ligament and the ASIS. This entrapment condition typically arises due to various factors, including trauma, pelvic tumors, prolonged leg/trunk hyperextension or standing, external compression from belts or snug attire, pregnancy, and weight gain. Additionally, it can be iatrogenic, resulting from certain surgical procedures. Clinically, it is marked by pain, paresthesia, dysesthesia, and numbness in the anterolateral thigh, from compression of the LFCN [2,3].

Diagnosis relies primarily on clinical evaluation, drawing from patient history and physical examination findings. Nevertheless, in cases where the diagnosis is less straightforward or appears atypical, diagnostic tests such as ultrasound, magnetic resonance imaging (MRI), computed tomography (CT) scans, and electrophysiological assessments can be valuable. These diagnostic measures help exclude other conditions that may mimic MP, including tumors, lumbar stenosis, disc herniation, and nerve root radiculopathy. Therefore, it is important to execute an extensive physical examination that allied with diagnostic imaging can help exclude those causes. Electrophysiological studies, in particular, can offer useful insights, as they can confirm the absence of motor nerve issues, which helps rule out root-related diseases [3,4].

The prognosis is generally favorable since most cases are self-limiting or respond to conservative treatment, such as lifestyle modification, pharmacological treatment and rehabilitation program, with physical agents, and soft tissue mobilization [3].

## Case presentation

We present the case of a 53-year-old man, overweight with a body mass index of 29, with no relevant medical history, who was a victim of a traffic accident in October 2023. It resulted in polytrauma with fracture of the 3rd-9th left costal arches and the anterior sternal plate, and fracture of the left radial styloid and the proximal phalanx of the right hallux. The treatment was conservative, with immobilization of the left arm and right hallux with a splint.

The patient was assessed by physiatry on the third day of hospitalization, after request for observation by general surgery. The main complaints of the patient were left thoracalgia with deep inspiration, controlled with the prescribed analgesia, and marked pain in the anterolateral aspect of the left thigh, described as burning and electric shock. He presented with bruising from the seatbelt on his chest and scattered abrasions on his lower and upper limbs. On the left thigh, he reported pain on palpation of the muscles in the lateral side of the proximal third, with no hematoma or associated deformities. The mobility of the left hip was preserved. There was tenderness to deep percussion, approximately two finger-breadth medial to the left ASIS. Tingling of the left thigh was elicited by leg extension at the hip. Sensory examination revealed dysesthesia to gentle rubbing along the anterolateral surface of the left thigh. He began physiatric treatment with soft tissue mobilization, desensitizing massage, and physical agents. Additionally, he initiated pregabalin, an anticonvulsant drug used to treat neuropathic conditions, at an initial dosage of 75 mg once daily, with gradual increase if necessary.

**Figure 1:**
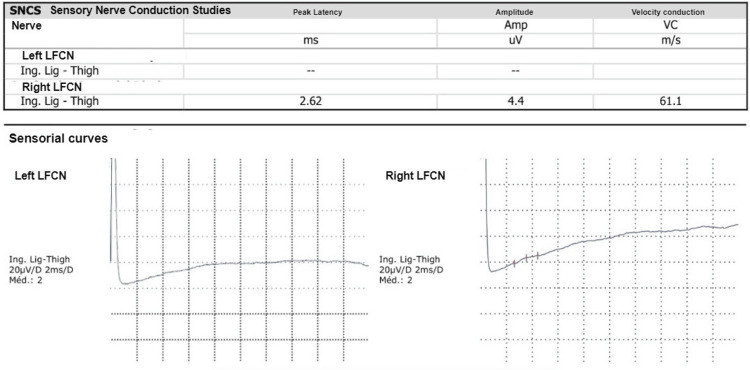
Complementary study: Electromyography of the left thigh LFCN: Lateral femoral cutaneous nerve

**Figure 2:**
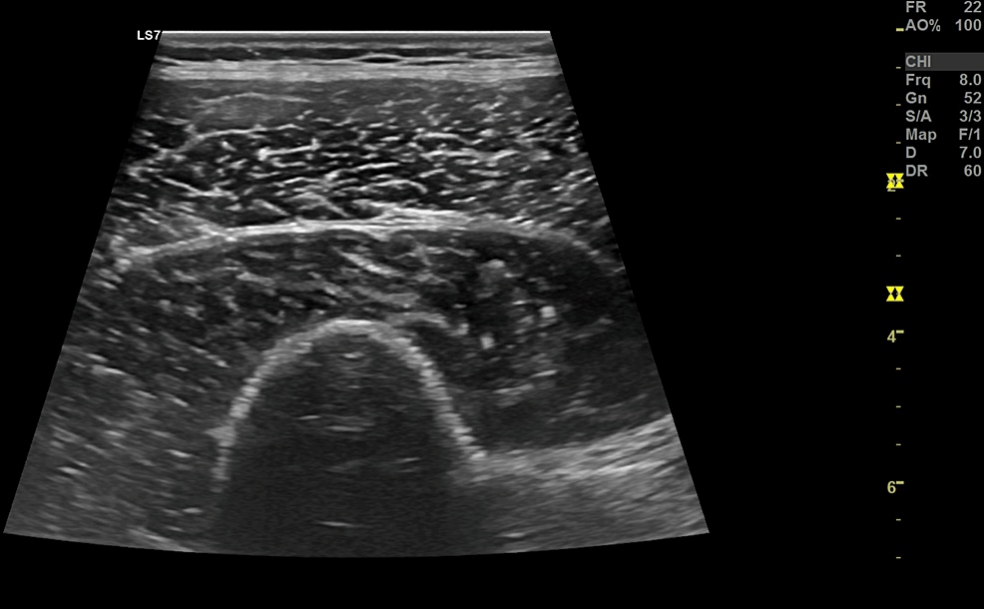
Complementary study: Soft-tissue ultrasound of the left thigh performed by Radiology No expansive lesions were observed in this topography, maintaining the normal fibrillar structure of the muscles. There was no fluid collection.

**Figure 3:**
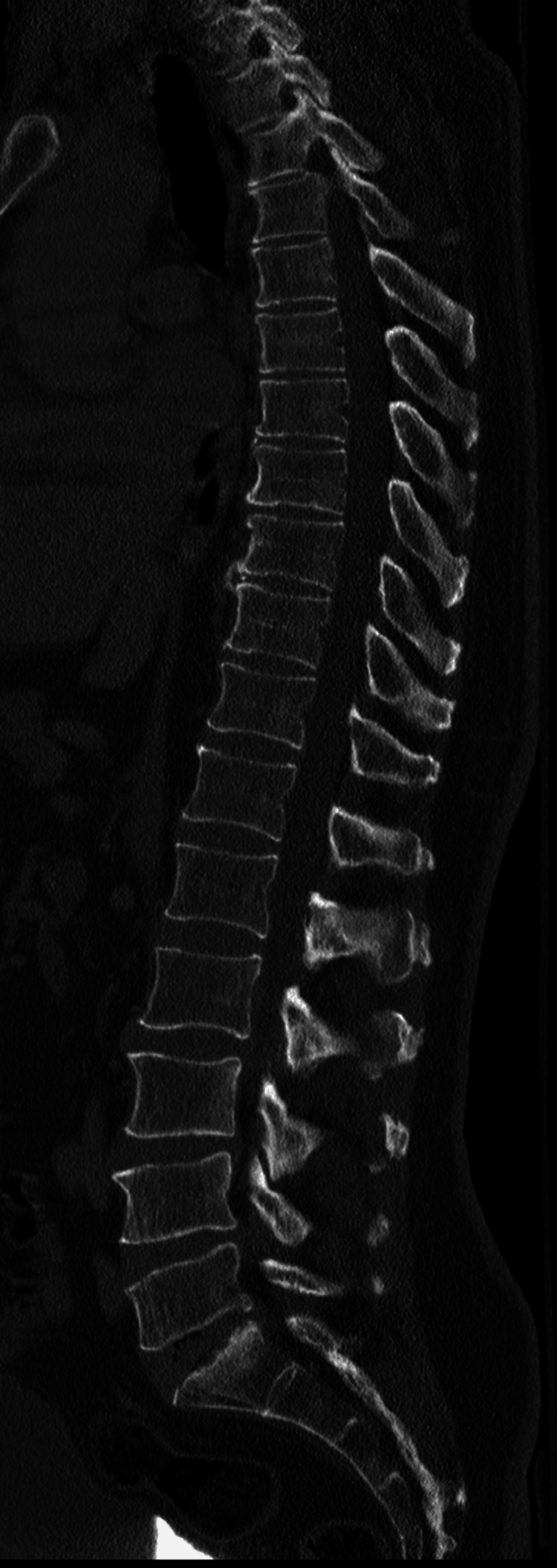
Complementary study: Dorsolumbar CT scan There were no obvious fractures of the cervical, dorsal, and lumbar vertebrae and no signs of obvious post-traumatic canal involvement. There were degenerative changes in the lumbosacral hinge.

In addition, during hospitalization, a diagnostic study of referred neuropathic pain to the left thigh was initiated. The complementary study showed axonal damage to the left femoral cutaneous nerve on electromyography (Figure 1) and no apparent alterations on soft tissue ultrasound of the left thigh performed by Radiology (Figure 2). A dorsolumbar CT scan was realized at admission, with minor degenerative alterations (Figure 3), excluding nerve root radiculopathy. A diagnosis of post-trauma MP was thus established, probably due to compression by the seat belt in the car accident.

Throughout the hospital stay, one week after starting treatment, the symptoms persisted and the neuropathic pain in the left thigh was the main complaint of the patient, despite the present fractures. Therefore, an interventional physiatrist's appointment was booked for the following day in order to try a more invasive treatment for pain control.

With the complement of the ultrasound, a large hematoma was seen, above the inguinal ligament, which could be compressing the lateral femoral cutaneous nerve. An ultrasound-guided drainage of 140cc of serosanguineous fluid was performed, with an in-plane approach, with reduction of the hypoechoic area (hematoma). After the procedure, the patient noticed immediate symptomatic improvement, with symptoms of residual dysesthesia on the anterolateral aspect of the left thigh. At three-month follow-up, the patient did not report worsening of the symptoms with no complications related to the therapeutic intervention.

## Discussion

The LFCN predominantly arises from the L2-L4 nerve roots and innervates the anterior and lateral aspects of the thigh from the inguinal ligament to the knee. Damage to the LFCN leads to a condition known as MP, characterized by sensations of tingling, reduced sensitivity, numbness, and pain, with no associated muscle weakness. Various factors, such as iatrogenic and traumatic causes, can contribute to this condition [2]. In this case, the most likely cause of LFCN compression was a hematoma after a seat belt injury in a car accident [5].

At times, the trauma mechanism is evident, as in this particular instance. However, there are situations where the trauma's origin remains unrecognized, leading to significant delays in patient referral. In cases such as those illustrated by Blake and Treble [6], abrasions spanning from the chest to the anterolateral hip region can serve as potential indicators, though these abrasions may not always be present. Consequently, it becomes beneficial to employ ultrasound analysis in these instances to explore the likelihood of a neuroma, hematoma, or fibrosis. Furthermore, applying pressure with the ultrasound probe over the injured area can induce symptoms (sonopalpation), which may further suggest a traumatic mechanism of injury [3].

The patient’s hematoma increased pressure on the iliopsoas section, with compression of the LFCN. If conservative treatments fail to yield improvement, neurological symptoms worsen, or if ultrasound clearly reveals nerve compression due to the hematoma, interventional techniques such as hematoma evacuation and percutaneous drainage need to be considered. The prognosis hinges on the duration and severity of nerve compression, but recovery typically takes from several days to a few months, with resolution of most cases within a six-month period.

## Conclusions

In the literature, cases of post-trauma MP including NCFL injury after a seat belt injury have been described, although rarely. This is a challenging and uncommon case, which highlights the importance of carrying out a complete objective examination combined with a rapid and accurate diagnosis, in order to begin treatment aimed at the pathology. It also emphasizes the importance of the clinical ultrasound in a physiatry consultation, which in this case was crucial to the treatment and probable prognosis of this patient.
